# Efficacy and safety of Huaier granules combined with chemotherapy for gastric cancer: A protocol for systematic review and meta-analysis

**DOI:** 10.1097/MD.0000000000021807

**Published:** 2020-08-21

**Authors:** Daorui Hou, Jian Xiong, Liangjun Yang, Lu Xiong

**Affiliations:** a Department of Traditional Chinese Medicine Oncology, The First People's Hospital of Xiangtan City, Xiangtan, Hunan Province, China; b Department of Oncology, Guang’anmen Hospital, Beijing, China; c Department of Gastroenterology, Tongde Hospital of Zhejiang Province, Hangzhou, Zhejiang Province, China.

**Keywords:** gastric cancer, Huaier, protocol, systematic review and meta-analysis

## Abstract

**Background:**

Huaier granules, the aqueous product of Huaier (*Trametes robiniophila Murr.*) extract, are a broad-spectrum anti-tumor drug and have been widely used for the treatment of gastric cancer (GC). The aim of this study is to systematically investigate the efficacy and safety of Huaier granules combined with chemotherapy in the treatment of GC.

**Methods:**

Three English databases and four Chinese databases will be searched from its inception to July 2020. Two methodological trained researchers will select the qualified studies for data extraction independently. Cochrane Risk of Bias tool will be used to assess the risk of bias of included studies. The RevMan 5.2 and stata 14.0 software will be applied for statistical analyses. Statistical heterogeneity will be computed by Cochrane *X*^2^ and *I*^2^ tests. Sensitivity analysis will be conducted to evaluate the stability of the results. The publication bias will be evaluated by funnel plots and Egger's test. The quality of evidence will be assessed by the GRADE system.

**Results:**

The results of our research will be published in a peer-reviewed journal.

**Conclusion:**

The conclusion of our systematic review will provide evidence to judge whether Huaier granules combined with chemotherapy is an effective intervention for patient with GC.

**OSF registration number:**

10.17605/OSF.IO/9BVJD.

## 1 Introduction

Gastric cancer (GC) is the fifth most frequently diagnosed cancer and the third leading cause of cancer death around the world.^[1,2]^ According to an epidemiological survey in 2018, there were 1,033,701 new cases of GC and 782,685 deaths related to GC worldwide.^[3]^ As a standard treatment for GC, chemotherapy-based treatment has increased the cure rate of about 10% in the treatment of GC in the past few years.^[4–6]^ However, the side effects and acquired drug resistance of chemotherapy restrict its application.^[7,8]^

Traditional Chinese medicine (TCM) has been effectively applied in treating malignant diseases for a long time in Eastern Asia.^[9–11]^ Clinical studies have confirmed that the combination of chemotherapy and TCM can improve the therapeutic effect of GC.^[12,13]^ Huaier granules, the aqueous product of Huaier (*Trametes robiniophila Murr.*), are one of the approved TCM by Chinese State Food and Drug Administration (SFDA) (Drug Approval Number: Z20000109). It has been used to treat liver cancer,^[14,15]^ breast cancer,^[16]^ lung cancer,^[17]^ and gastrointestinal cancer.^[18,19]^ Recent research has shown that Huaier granules can modulate innate immunity through the release of cytokines and the generation of reactive oxygen species and NO.^[20]^ Furthermore, it exerts anti-tumor responses by inducing cell cycle arrest at the G0/G1 checkpoint and inhibiting tumor angiogenesis.^[21]^

Recently, the using of Huaier granules combined with chemotherapy for treating GC has attracted increasing worldwide interest.^[22–24]^ Although several epidemiologic studies were performed for its efficiency with gastric cancer, no consensus conclusion has been reached yet.^[19]^ The efficacy and safety of Huaier granules combined with chemotherapy in the treatment for GC remain controversial. Hereby, we will systematically review current available randomized controlled trials (RCTs) to objective comment the efficacy and safety of Huaier granules during chemotherapy in patients with GC. It may provide more relevant information for clinician in clinical practice.

## 2 Methods and analysis

### 2.1 Study registration

This work has been registered at Open Science Framework (OSF, https://osf.io/), an open source project management that helps in the design of studies. The registration DOI of this study is 10.17605/OSF.IO/9BVJD. The protocol of our meta-analysis will be conducted followed the guideline of the Preferred Reporting Items for Systematic Review and Meta-Analysis Protocols (PRISMA-P) recommendations.^[25]^

### 2.2 Eligibility criteria

#### 2.2.1 Study design

Randomized controlled trials (RCTs) which used a combination of Huaier granules and chemotherapy as treatment measures will be eligible, while laboratory studies, qualitative studies, or observational study will be excluded in the research. There are no limitations on language and publication status.

#### 2.2.2 Types of participants

We will include RCTs on participants who are diagnosed as GC. The race, sex, age, severity, and duration are not restricted.

#### 2.2.3 Types of interventions

Interventions to be reviewed are Huaier granules combination with chemotherapy to treat the GC. The control intervention could be any management in patients with GC.

### 2.3 Outcomes

The primary outcomes of this review will focus on overall survival (OS) and progression-free survival (PFS). The secondary outcomes included overall response rate (ORR), disease control rate (DCR), quality of life improved rate (QIR), and adverse events.

### 2.4 Search strategy

To ascertain the relevant literature, 3 English databases including PubMed, Embase, Cochrane Library Central Register of Controlled Trials, and 4 Chinese databases including China National Knowledge Infrastructure (CNKI) database, Wanfang Data Knowledge Service Platform, Chinese Scientific Journal Database (VIP), China Biology Medicine Disc (Sino Med) will be searched from its inception to July 2020. In addition, Google scholar, Bing scholar, and Baidu scholar will be retrieved to find out other related literature. Moreover, the Chinese Clinical Trial Registry (ChiCTR) and ClinicalTrials.gov will also be searched. We will also search in OpenGrey.eu. website for potential gray literature. Two authors (DH and JX) will search and screen all the citations independently. An example of search strategy for PubMed database that combines MeSH terms and free words will be adopted. The search strategy was as follows:

#1 Search: (“Stomach Neoplasms”[Mesh]) OR ((((((((((((((((((Neoplasm, Stomach[Title/Abstract]) OR (Stomach Neoplasm[Title/Abstract])) OR (Neoplasms, Stomach[Title/Abstract])) OR (Gastric Neoplasms[Title/Abstract])) OR (Gastric Neoplasm[Title/Abstract])) OR (Neoplasm, Gastric[Title/Abstract])) OR (Neoplasms, Gastric[Title/Abstract])) OR (Cancer of Stomach[Title/Abstract])) OR (Stomach Cancers[Title/Abstract])) OR (Gastric Cancer[Title/Abstract])) OR (Cancer, Gastric[Title/Abstract])) OR (Cancers, Gastric[Title/Abstract])) OR (Gastric Cancers[Title/Abstract])) OR (Stomach Cancer[Title/Abstract])) OR (Cancer, Stomach[Title/Abstract])) OR (Cancers, Stomach[Title/Abstract])) OR (Cancer of the Stomach[Title/Abstract])) OR (Gastric Cancer, Familial Diffuse[Title/Abstract]))

#2 Search: (“huaier” [Supplementary Concept]) OR (((((Trametes robiniophila Murr[Title/Abstract]) OR (Auricularia auricula[Title/Abstract])) OR (Jin Ke[Title/Abstract])) OR (Jinke[Title/Abstract])) OR (Jew Ear Parasitized Granula[Title/Abstract]))

#3 Search: (((((((((randomized controlled trial[Title/Abstract]) OR RCT[Title/Abstract]) OR random[Title/Abstract]) OR randomly[Title/Abstract]) OR random allocation[Title/Abstract]) OR allocation[Title/Abstract]) OR randomized control trial[Title/Abstract]) OR controlled clinical trial[Title/Abstract]) OR clinical trial[Title/Abstract]) OR clinical study[Title/Abstract]

#1 and #2 and #3

### 2.5 Selection of studies

**Figure 1:**
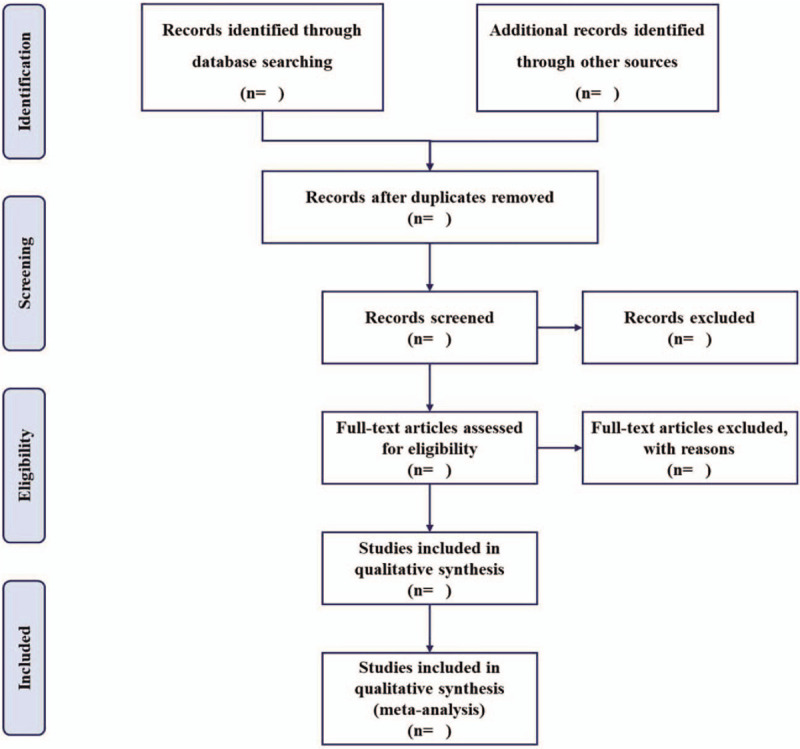
Flow chart of study selection.

The electronic citations extracted out from the above databases will be managed by EndNote X9.0 (Stanford, Connecticut, https://endnote.com). Two methodological trained investigators (DH and JX) will independently review all identified data based upon the exclusion and inclusion criteria and remove duplicate literature. Titles, abstracts, and full-text articles will be screened and data will be extracted independently by those reviewers. Any divergences between 2 investigators will be solved through discussion with another investigator (LY). We will note reasons for all excluded studies. A PRISMA flow chart (Fig. 1) will be drawn to present the whole process of study selection.^[25]^

### 2.6 Extraction and management of data

Two investigators (DH and JX) will extract relevant data independently with the standardized sheet recommended by the Cochrane Handbook of Systematic Reviews of Interventions. The data of those qualified articles will be export to Microsoft Excel, which includes the first author, published year, study location, study design, inclusion/exclusion criteria, pathological type, sample size, participants’ baseline characteristics, intervention characteristics, control, outcome data, and adverse events. For articles with incomplete or uncertain data, the authors will be contacted for complete data whenever possible. If there is any dispute in the data extraction process, it will be submitted to a third researcher (LX) for processing.

### 2.7 Assessment of risk of bias in included studies

The Cochrane collaboration's tool, an established and reliable tool for assessing the risk of bias, will be used in studies evaluate the risk of bias for each study by two independent reviewers (DH and LY).^[26]^ In this tool, the risk of bias of a trial is evaluated through 7 items, include random sequence generation (selection bias), allocation concealment (selection bias), blinding of participants and personnel (performance bias), blinding of outcome assessment (detection bias), incomplete outcome data (attrition bias), selective reporting (reporting bias), other bias. The assessment will be classified as “Low risk,” “High risk,” or “Unclear risk.” Disagreements between the 2 reviewers will be resolved by discussion of all reviewers.

### 2.8 Synthesis of data

RevMan 5.2 (Cochrane, London, UK) and Stata 14.0 software (Stata Corporation, College Station, TX, USA) will be applied for statistical analyses. For dichotomous variables, the risk ratio (RR) will be applied to analyze. For continuous variables, a mean difference (MD) or a standard mean difference (SMD) will be used for analysis. MD will be used when the treatment outcome was measured by the same scale. SMD will be used when the treatment outcome was measured by different scales in different studies. The confidence intervals (CI) for both dichotomous and continuous variables will be set to 95%.

### 2.9 Assessment of heterogeneity

Statistical heterogeneity will be computed by Cochrane *X*^2^ and *I*^2^ tests.^[27]^ 75%, 50%, or 25% of *I*^2^ statistics indicate high, medium, and low heterogeneity, respectively. The statistical heterogeneity is considered substantial when *P* < .05 and *I*^2^ > 50% and the random-effect model will be applied to pool data. In the case of high heterogeneity, we will conduct subgroup analysis according to the region of the studies, age, stage of the subjects, types of treatments, and different outcomes. We will evaluate the credibility of the subgroup analysis in term of the guidance.^[28]^ If there are enough researches, meta-regression will be performed to clarify the source of heterogeneity. If there is no significant heterogeneity (*P* > .05 and *I*^2^ < 50%), then the fixed-effect model will be used to calculate the effect size. If quantitative synthesis is not appropriate due to substantial heterogeneity, then systematic review will be conducted and the results will be displayed in tables and figures.

### 2.10 Sensitivity analysis

To ensure the stability of the results, a sensitivity analysis will be performed for the outcomes. We will exclude each study included in the analysis one by one. Then we will reanalyze and compile the data and compare the difference between the reobtained effects and the original effects. If there is one or more very large study, we will repeat the analysis excluding them to determine how much they dominate the results.

### 2.11 Assessment of reporting biases

The publication bias will be evaluated by funnel plots when >10 studies are included. Egg regression and Begger tests will be utilized to detect the funnel plot asymmetry.^[29]^*P* < .05 is considered to have publication bias. In the case of asymmetric funnel plot, subgroup analysis or sensitivity analysis will be performed to investigate possible causes.^[30]^

### 2.12 Grading the quality of evidence

We will assess the quality of evidence using the Grading of Recommendations Assessment, Development and Evaluation (GRADE), a widely used tool in evaluating the quality of assessment.^[31]^ The quality of evidence will be assorted into “high,” “moderate,” “low,” and “very low” quality.

### 2.13 Patient and public involvement

Patient and public were not involved in this study.

### 2.14 Ethics and dissemination

Ethical approval will not be required for this systematic review because the data used are not linked to individual patient. The results of this review will be disseminated by being published in a peer-reviewed journal.

## 3 Discussion

GC, a common cancer with high morbidity and mortality worldwide, requires continuous exploration for new treatment methods and concepts.^[32]^ Huaier granule is a famous Chinese patent medicine for treating GC in clinical practice, and a series of clinical studies have been conducted on it.^[33–35]^ However, there is no systematic review related to Huaier granules combined with chemotherapy for GC published. In this study, we will conduct systematic review and meta-analysis to provide more evidence on the effectiveness and safety for Huaier granules combined with chemotherapy. These findings may provide more guidance for clinicians in the treatment of GC.

### 3.1 Amendments

If amendments are needed, we will update our protocol to include any changes in the whole process of research.

## Author contributions

**Conceptualization:** Lu Xiong.

**Data curation:** Daorui Hou, Jian Xiong, Liangjun Yang.

**Formal analysis:** Liangjun Yang, Lu Xiong.

**Funding acquisition:** Lu Xiong.

**Investigation:** Daorui Hou, Jian Xiong, Liangjun Yang.

**Methodology:** Daorui Hou, Liangjun Yang, Lu Xiong.

**Project administration:** Lu Xiong.

**Resources:** Daorui Hou, Jian Xiong, Liangjun Yang.

**Software:** Daorui Hou, Jian Xiong, Liangjun Yang.

**Supervision:** Lu Xiong.

**Writing – original draft:** Daorui Hou, Jian Xiong.

**Writing – review & editing:** Daorui Hou, Jian Xiong, Liangjun Yang, Lu Xiong.

## References

[1] Thrift AP El-Serag HB Burden of gastric cancer. Clin Gastroenterol Hepatol 2020;18:534–42. 31362118 10.1016/j.cgh.2019.07.045 PMC8859863

[2] Venerito M Vasapolli R Rokkas T Gastric cancer: epidemiology, prevention, and therapy. Helicobacter 2018;23:e12518. 30203589 10.1111/hel.12518

[3] Bray F Ferlay J Soerjomataram I Global cancer statistics 2018: GLOBOCAN estimates of incidence and mortality worldwide for 36 cancers in 185 countries. CA Cancer J Clin 2018;68:394–424. 30207593 10.3322/caac.21492

[4] Salati M Orsi G Smyth E Gastric cancer: translating novels concepts into clinical practice. Cancer Treat Rev 2019;79:1–9. 10.1016/j.ctrv.2019.101889 31445415

[5] Wagner AD Syn NL Moehler M Chemotherapy for advanced gastric cancer. Cochrane Database Syst Rev 2017;8:Cd004064. 28850174 10.1002/14651858.CD004064.pub4 PMC6483552

[6] Ter Veer E Haj Mohammad N van Valkenhoef G The efficacy and safety of first-line chemotherapy in advanced esophagogastric cancer: a network meta-analysis. J Natl Cancer Inst 2016;108:1–3. 10.1093/jnci/djw166 27576566

[7] Drews RE Shulman LN Update in hematology and oncology. Ann Intern Med 2010;152:655–62. 20410447 10.7326/0003-4819-152-10-201005180-00244

[8] Takashima A Shirao K Hirashima Y Sequential chemotherapy with methotrexate and 5-fluorouracil for chemotherapy-naive advanced gastric cancer with disseminated intravascular coagulation at initial diagnosis. J Cancer Res Clin Oncol 2010;136:243–8. 19727819 10.1007/s00432-009-0655-8 PMC11828333

[9] Hsiao WL Liu L The role of traditional Chinese herbal medicines in cancer therapy--from TCM theory to mechanistic insights. Planta Med 2010;76:1118–31. 20635308 10.1055/s-0030-1250186

[10] Xiang Y Guo Z Zhu P Traditional Chinese medicine as a cancer treatment: modern perspectives of ancient but advanced science. Cancer Med 2019;8:1958–75. 30945475 10.1002/cam4.2108 PMC6536969

[11] Xu W Towers AD Li P Traditional Chinese medicine in cancer care: perspectives and experiences of patients and professionals in China. Eur J Cancer Care (Engl) 2006;15:397–403. 16968323 10.1111/j.1365-2354.2006.00685.x

[12] Hong X-C Liang Q-L Luo X-B Clinical study of XiangShaLiuJunZi decoction combined with S-1 as maintenance therapy for stage III or IV gastric carcinoma and colorectal carcinoma. Medicine (Baltimore) 2020;99:1–6. 10.1097/MD.0000000000020081 PMC7440293 32384478

[13] Zhang X Yuan Y Xi Y Cinobufacini injection improves the efficacy of chemotherapy on advanced stage gastric cancer: a systemic review and meta-analysis. Evid Based Complement Alternat Med 2018;2018:1–2. 10.1155/2018/7362340 PMC6142757 30254688

[14] Zhao GS Liu Y Zhang Q Transarterial chemoembolization combined with Huaier granule for the treatment of primary hepatic carcinoma: safety and efficacy. Medicine (Baltimore) 2017;96:1–4. 10.1097/MD.0000000000007589 PMC5521939 28723799

[15] Chen Q Shu C Laurence AD Effect of Huaier granule on recurrence after curative resection of HCC: a multicentre, randomised clinical trial. Gut 2018;67:2006–16. 29802174 10.1136/gutjnl-2018-315983

[16] Zhang Y Wang X Chen T Efficacy of Huaier granule in patients with breast cancer. Clin Transl Oncol 2019;21:588–95. 30276759 10.1007/s12094-018-1959-4

[17] Chen Y Wu H Wang X Huaier Granule extract inhibit the proliferation and metastasis of lung cancer cells through down-regulation of MTDH, JAK2/STAT3 and MAPK signaling pathways. Biomed Pharmacother 2018;101:311–21. 29499405 10.1016/j.biopha.2018.02.028

[18] Yao J-G Han S-L Zhu G-B Effect of postoperative adjuvant chemotherapy combined with Huaier Granule for Stage III Gastric Cancer. Bull Chin Cancer 2003;12:606–8.

[19] Ma Y Wang C Zhang Q The effects of polysaccharides from Auricularia auricula (Huaier) in adjuvant anti-gastrointestinal cancer therapy: a systematic review and network meta-analysis. Pharmacol Res 2018;132:80–9. 29673791 10.1016/j.phrs.2018.04.010

[20] Schepetkin IA Quinn MT Botanical polysaccharides: macrophage immunomodulation and therapeutic potential. Int Immunopharmacol 2006;6:317–33. 16428067 10.1016/j.intimp.2005.10.005

[21] Wang X Zhang N Huo Q Anti-angiogenic and antitumor activities of Huaier aqueous extract. Oncol Rep 2012;28:1167–75. 22895629 10.3892/or.2012.1961 PMC3583466

[22] Song X Li Y Zhang H The anticancer effect of Huaier (Review). Oncol Rep 2015;34:12–21. 25955759 10.3892/or.2015.3950

[23] Pan J Yang C Jiang Z Trametes robiniophila Murr: a traditional Chinese medicine with potent anti-tumor effects. Cancer Manag Res 2019;11:1541–9. 30863164 10.2147/CMAR.S193174 PMC6389013

[24] Li C Wang X Chen T Trametes robiniophila Murr in the treatment of breast cancer. Biomed Pharmacother 2020;128:1–0. 10.1016/j.biopha.2020.110254 32480220

[25] Moher D Shamseer L Clarke M Preferred reporting items for systematic review and meta-analysis protocols (PRISMA-P) 2015 statement. Syst Rev 2015;4:1–9. 25554246 10.1186/2046-4053-4-1 PMC4320440

[26] Higgins JP Altman DG Gøtzsche PC The Cochrane Collaboration's tool for assessing risk of bias in randomised trials. BMJ 2011;343:1–9. 10.1136/bmj.d5928 PMC3196245 22008217

[27] Higgins JP Thompson SG Quantifying heterogeneity in a meta-analysis. Stat Med 2002;21:1539–58. 12111919 10.1002/sim.1186

[28] Sun X Briel M Walter SD Is a subgroup effect believable? Updating criteria to evaluate the credibility of subgroup analyses. BMJ 2010;340:850–4. 10.1136/bmj.c117 20354011

[29] Peters JL Sutton AJ Jones DR Contour-enhanced meta-analysis funnel plots help distinguish publication bias from other causes of asymmetry. J Clin Epidemiol 2008;61:991–6. 18538991 10.1016/j.jclinepi.2007.11.010

[30] Shuster JJ Higgins JPT Green S Cochrane handbook for systematic reviews for interventions, Version 5.1. 0, published 3/2011. Res Synth Methods 2011;2:126–30.

[31] Atkins D Best D Briss PA Grading quality of evidence and strength of recommendations. BMJ 2004;328:1–8. 15205295 10.1136/bmj.328.7454.1490 PMC428525

[32] Rawla P Barsouk A Epidemiology of gastric cancer: global trends, risk factors and prevention. Prz Gastroenterol 2019;14:26–38. 30944675 10.5114/pg.2018.80001 PMC6444111

[33] Tang Q Zhan X-y Liu J-h Effect of Huaier granule in treatment for advanced gastric cancer in 47 elderly cases. Bull Chinese Cancer 2006;15:137–8.

[34] Liu C Sun X-h The impact of Huaier particles on T lymphocyte subsets in peripheral blood of 30 patients with advanced gastric cancer. Med Recapitulate 2013;19:1292–3.

[35] De-lin J Da-hai M Effect of Huaier granule on immunity and quality of life in patients with gastric cancer undergoing postoperative concurrent radiochemotherapy. China Cancer 2010;19:73–6.

